# Glycated hemoglobin versus oral glucose tolerance test in the identification of subjects with prediabetes in Qatari population

**DOI:** 10.1186/s12902-019-0412-1

**Published:** 2019-08-22

**Authors:** Saadallah Iskandar, Ayman Migahid, Dalia Kamal, Osama Megahed, Ralph A. DeFronzo, Mahmoud Zirie, Amin Jayyousi, Mahmood Al Jaidah, Muhammad Abdul-Ghani

**Affiliations:** 1 Qatar Petroleum, Doha, Qatar; 2 Academic Health System, Hamad General Hospital, PO Box 3050, Doha, Qatar; 3 Diabetes Division, University of Texas Health Science Center, San Antonio, TX USA

**Keywords:** Prediabetes, Type 2 diabetes, HbA1c, OGTT

## Abstract

**Background:**

Subjects with prediabetes are at increased risk of future T2DM and cardiovascular disease (CVD) compared to NGT individuals. The OGTT (FPG = 100–125 and 2 h-PG = 140–199 mg/dl) and HbA1c 5.7–6.4% have been used to diagnose subjects with prediabetes. In the present study, we compared the ability of the OGTT and HbA1c to identify Qatari subjects with prediabetes.

**Methods:**

Four hundred forty six subjects without a history of T2DM received 75-g OGTT and measurement of HbA1c. The incidence of prediabetes in this cohort according to OGTT criteria was compared to that of HbA1c criteria.

**Results:**

The agreement between the OGTT and HbA1c in identifying subjects with prediabetes in Qatari subjects was poor, though significant (k = 015, *p* < 0.0001). Only 56% of participants had prediabetes or NGT according to OGTT and HbA1c. The disagreement between OGTT and HbA1c in diagnosing prediabetes was primarily due to low sensitivity of HbA1c. Moreover, subjects with prediabetes diagnosed with the OGTT have more severe metabolic profile than prediabetic subjects diagnosed with HbA1c. Lastly, more subjects with the metabolic syndrome were identified with OGTT (60%) criteria than with the HbA1c (49%), *p* < 0.0001.

**Conclusion:**

These results demonstrate subjects with prediabetes diagnosed with OGTT have more severe metabolic risk than those diagnosed with HbA1c, and more likely to have greater risk of progression to T2DM.

## Background

The prevalence of type 2 diabetes mellitus (T2DM) is increasing at epidemic rates worldwide and the diabetes epidemic is even more widespread in the Middle East [1–3]. The prevalence of T2DM exceeds 20% in many Gulf countries and an additional ~ 20% of the population is at increased future risk, i.e. prediabetes [4, 5]. In Qatar, 16.5% manifest T2DM and approximately similar number has prediabetes [4]. Thus, approximately one third of adults Qatari individuals have prediabetes or established diabetes. Both lifestyle change and pharmacotherapy have been shown to reduce the risk of progression to T2DM in high risk individuals [6–8]. Thus, identification of high risk individuals is a key for diabetes prevention and restraining the epidemic spread of the disease [9].

Because subjects with impaired fasting glucose (IFG) and/or impaired glucose tolerance (IGT) manifest greater risk of progression to T2DM than subjects with normal glucose tolerance (NGT) [10], both IFG and IGT are considered to be prediabetes states by the American Diabetes Association [11], and all intervention studies which have assessed the efficacy of various intervention strategies have recruited subjects with IGT, IFG, or IFG/IGT [6–8]. Because the diagnosis of IGT requires an oral glucose tolerance test (OGTT), the International Diabetes Federation has suggested that the diagnosis of prediabetes be made based upon the HbA1c since it does not require an OGTT and can be measured under non-fasting conditions [12]. HbA1c between 5.7 to 6.4% has been identified as the diagnostic range of prediabetes. Studies which have compared the ability of HbA1c versus OGTT to identify subjects with prediabetes [13–18] have reported discordance between the two methods. Further, the degree of agreement between the two methods varies amongst various ethnic groups [15, 17]. Accurate identification of high risk individuals is essential for any intervention program, since it lowers the number of subjects enrolled in the intervention program and increases the cost effectiveness of the intervention program. The state of Qatar is considering a National Diabetes Strategy for diabetes prevention based upon the identification of high risk individuals using the HbA1c. Because of the disagreement between the diagnosis of prediabetes based upon HbA1c criteria versus the OGTT and the ethnic dependence of the concordance between both methods, it is important to determine the optimal test and the appropriate cut points for detection of subjects with prediabetes in Qatari population. The aim of the present study is to compare the HbA1c versus the OGTT in the identification of Qatari subjects with prediabetes and the underlying metabolic abnormalities associated with prediabetes.

## Methods

The present study was a retrospective analysis of data collected in healthy subjects, without a history of T2DM, who are seeking employment opportunity at Qatar Petroleum. Each subject seeking employment at Qatar Petroleum receives a comprehensive physical examination, lab work and 75-g OGTT to ensure that he is in a good health. Subjects who fulfilled the following criteria were included in the analysis: 1) Arabic origin; (2) no history of T2DM; (3) had HbA1c < 6.5%, and FPG < 126 mg/dl and 2-h plasma glucose < 200 mg/dl; (4) general good health as indicated by CBC, blood chemistry and thyroid function.

Four hundred forty six subjects were eligible for the analysis. Medical history, physical examination, blood chemistries, lipid profile, and HbA1c were measured. The clinical and metabolic characteristics of the subjects are shown in Table 1. None of the subjects were taking any medications known to affect glucose tolerance.

**Table 1:** Baseline characteristics of subjects with prediabetes and NGT

	NGT	IFG/IGT	*P* Value
Number	150	296	
Age (years)	45 ± 1	44 ± 1	NS
Gender (females)	21 (14%)	55 (19%)	NS
BMI (kg/m^2^)	30.3 ± 0.4	29.6 ± 0.3	NS
Waist (cm)	99.6 ± 0.7	101.3 ± 0.8	NS
Positive Family History	62%	64%	NS
FPG (mM)	5.22 ± 0.04	5.94 ± 0.04	< 0.0001
2-h PG (mM)	5.78 ± 0.08	7.60 ± 0.14	< 0.0001
HbA1c (%)	5.52 ± 0.03	5.67 ± 0.02	< 0.0001
Systolic Blood Pressure	125 ± 1	129 ± 1	0.01
Total Cholesterol (mM)	4.7 ± 0.1	4.9 ± 0.1	NS
Triglycerides (mM)	1.34 ± 0.07	1.66 ± 0.08	0.04
HDL (mM)	1.11 ± 0.03	1.09 ± 0.06	NS

*BMI* Body mass index, *FPG* fasting plasma glucose, *2-h PG* 2-h plasma glucose concentration

The OGTT was performed in the morning after a 10–12 h overnight fast. Blood samples were collected before and 2 h after drinking 75 g glucose in 300 ml of orange flavored solution. Plasma glucose concentration and serum insulin were measured during the fasting state and at 2-h.

The study protocol was approved by Hamad Medical Corporation IRB board (HMC Ethical committee) and informed written consent was obtained from participants prior to enrollment.

## Analytical methods

Glucose was measured with the glucose oxidase method. The variability of the measurement was 2.7%. HbA1c was measured by high pressure liquid chromatography (HPLC) (Knauer HPLC, Advanced Scientific Instruments, Germany). Blood pressure was measured with Digital Omni which is user independent, in the sitting position after 10 min of rest and represents the mean of 3 values.

## Calculations and statistical analysis

Definitions: prediabetes was designed as HbA1c = 5.7–6.4%, and subjects with HbA1c < 5.7% were considered having normal glucose tolerance. The ADA criteria of prediabetes were used to diagnosed prediabetes with OGTT (i.e. FPG = 100–125 mg/dl and/or 2-h plasma glucose =140–199 mg/dl).

Insulin sensitivity and insulin secretion indices were calculated with Homeostatsis Model Assessment, HOMA-IR and HOMA-B as previously described [19]. Beta cell function was measured as the product of insulin secretion (measured with HOMA-B) and (1/HOMA-IR). The ATPIII criteria for the metabolic syndrome [20] were utilized to diagnose the presence of the metabolic syndrome.

To estimate the future T2DM risk of study participants, the multivariate logistic prediction model described by Stern et al. [21] was utilized. This model estimates the 7–8 year diabetes risk and has been validated in multiple prospective population studies with 80% sensitivity and 78% specificity.

The κ-coefficient was used to test for agreement between A1C categorization of subjects and OGTT-based diagnoses of normal glucose tolerance and prediabetes.

## Results

One hundred fifty out of 446 participants (34%) had NGT and 296 subjects (66%) had prediabetes (IFG and/or IGT) according to the ADA criteria [22]. Table 1 presents the baseline characteristics of subjects with prediabetes and NGT. Both groups were comparable in age, gender, BMI and family history of T2DM. NGT subjects had a significantly lower HbA1c, FPG and 2-h plasma glucose concentration compared to subjects with prediabetes.

**Fig. 1:**
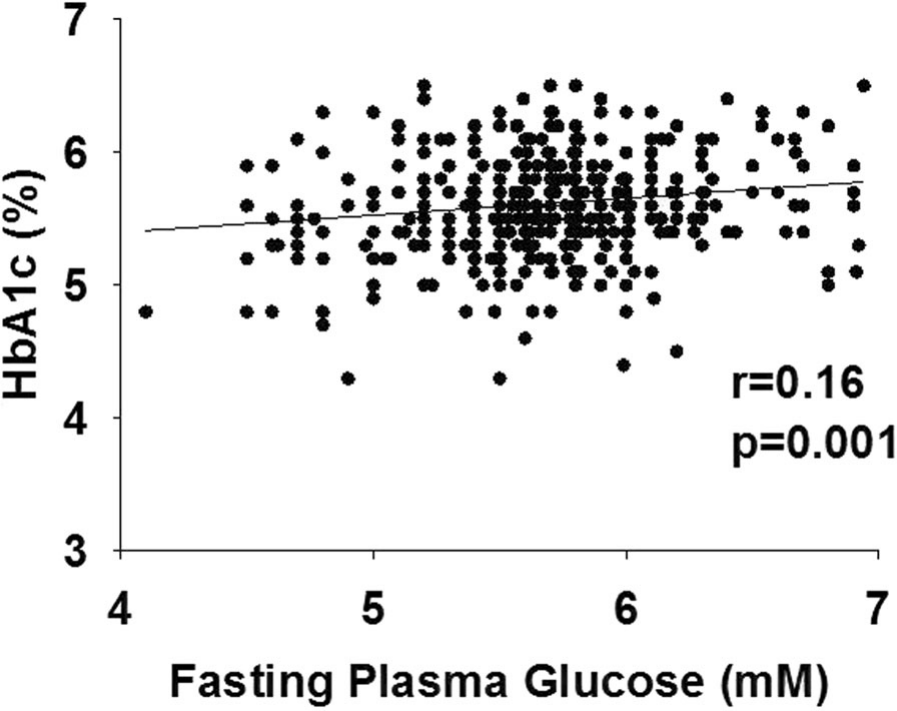
Relationship between HbA1c and the fasting plasma glucose concentration in subjects with NGT and prediabetes

**Fig. 2:**
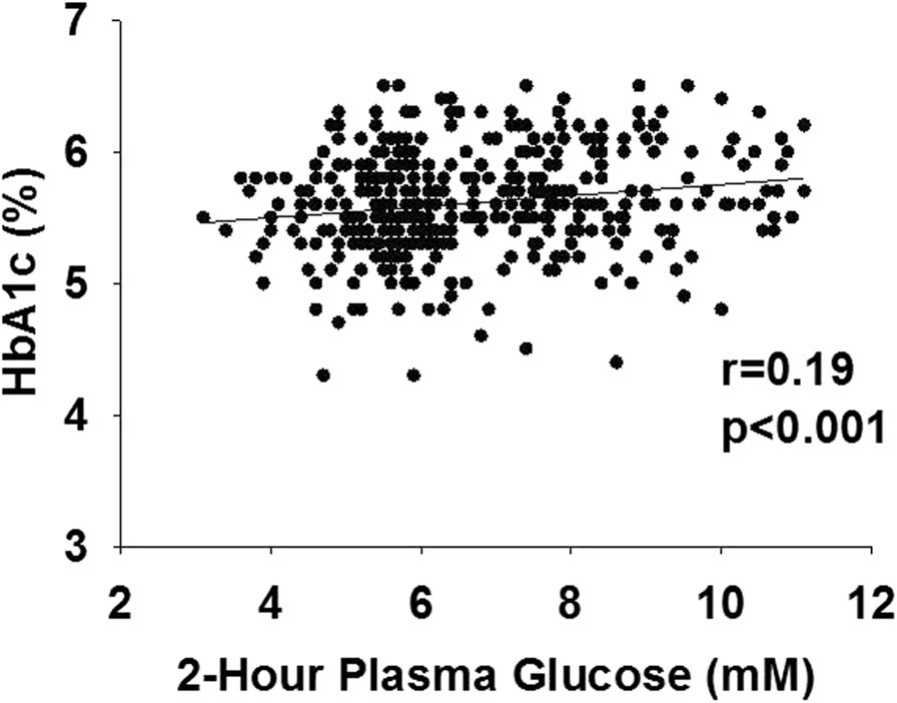
Relationship between HbA1c and 2-h plasma glucose concentration in subjects with NGT and prediabetes

Two hundred subjects (45%) had an HbA1c = 5.7–6.4% and, therefore, were considered to have prediabetes according to this parameter (*p* = 0.0007 versus OGTT criteria). The other 246 participants had HbA1c < 5.7% and, therefore, were considered normal according to HbA1c criteria (*p* < 0.001 versus OGTT criteria). There was large discordance in the diagnosis of prediabetes and NGT diagnosed with OGTT versus HbA1c criteria (Table 2). Only 150 subjects were diagnosed with prediabetes according to both criteria and 100 subjects had NGT according to both criteria. Thus, 196 subjects (44%) had the diagnosis of prediabetes or NGT according to one criteria and the opposite diagnosis according to the other criteria. Therefore, the agreement between OGTT and HbA1c in diagnosing glucose tolerance was low, though significant (k = 0.15, *p* < 0.0001). Further, there was a weak, though significant, correlation between the HbA1c and FPG (r = 0.16, *p* = 0.01) (Fig. 1) and between the HbA1c and 2-h plasma glucose concentration (r = 0.19, *p* < 0.001) (Fig. 2).

**Table 2:**
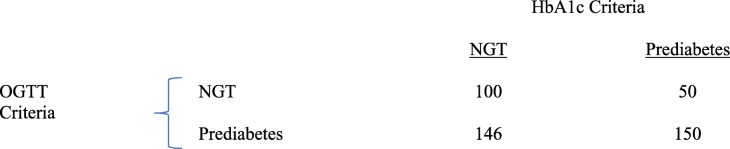
Prevalence of subjects with NGT and prediabetes diagnosed with OGTT and HbA1c

Previous studies [13, 18] have reported interaction between BMI and the concordance between HbA1c and OGTT in the diagnosis of glucose tolerance. In the present study we failed to observe any interaction between the diagnosis of glucose tolerance and either BMI or gender. The concordance between the two sets of criteria was 51, 53 and 59% in subjects with BMI < 27, 27–30 and > 30 kg/m^2^, respectively (*p* = NS), while the concordance was 50% in females and 57% in males (*p* = NS).

Because HbA1c and OGTT defined distinct populations of high risk individuals, we have compared the metabolic profile of subjects classified based upon OGTT versus HbA1c. Impaired beta cell function is the principal factor responsible for the increased future T2DM risk [23]. Further, subjects at risk of T2DM manifest impaired beta cell function long before T2DM become evident. Therefore, we compared beta cell function in subjects diagnosed with and without prediabetes with HbA1c and OGTT criteria. Compared to NGT, subjects with prediabetes had significantly lower beta cell function whether prediabetes was diagnosed with HbA1c (by 11%, *p* < 0.01) or with OGTT (by 37%, *p* < 0.0001) (Table 3). However, beta cell function in subjects diagnosed as NGT with the HbA1c was significantly lower (by 21%, *p* < 0.0001) than in NGT subjects diagnosed with OGTT. Conversely, subjects diagnosed as prediabetes with HbA1c had significantly higher beta cell function (by 13%, *p* < 0.0001) than subjects diagnosed with prediabetes with the OGTT. Moreover, beta cell function in subjects with prediabetes diagnosed with OGTT criteria but with HbA1c < 5.7% (i.e., NGT according to HbA1c criteria) was comparable to that in subjects with prediabetes according to both sets of criteria. Conversely, subjects with prediabetes according to HbA1c criteria but with NGT according to OGTT had comparable beta cell function to that of NGT subjects diagnosed with both sets of criteria (Table 4). Further, the metabolic profile of subjects with prediabetes diagnosed with the OGTT only was more severe than the profile of those diagnosed with HbA1c alone (Table 4). Consistent with this, the probability of future T2DM estimated with the Stern model in subjects diagnosed with prediabetes by the OGTT was 2.2-fold greater than in NGT subjects, compared to only 49% higher in subjects with prediabetes diagnosed with the HbA1c (last row Table 4).

**Table 3:** Beta cell function (measured with HOMA Model Assessment [19], and T2DM risk measured with the risk score developed by Stern et al. [21] and prevalence of the metabolic syndrome (according to ATPIII criteria [20] in NGT and subjects with prediabetes diagnosed with the OGTT and HbA1c

	HbA1c Criteria			OGTT Criteria		
	Normal	Prediabetes	*P*-Value	Normal	Prediabetes	*P*-Value
Number	246	200		150	296	
Beta Cell Function	42 ± 1	37 ± 1	0.001	53 ± 2	33 ± 1	< 0.0001
% With MS	44	49	NS	20	60	< 0.0001
Future T2DM Risk Score	12.9 ± 0.8	19.3 ± 1.2	< 0.0001	8.8 ± 0.5	19.3 ± 1.1	< 0.0001

*MS* metabolic syndrome

**Table 4:** Beta cell function (measured with HOMA Model [19] and prevalence of metabolic syndrome according to ATPIII criteria [20] in subjects discordant and concordant for the diagnosis of glucose tolerance with OGTT and HbA1c

	Normal by Both Criteria	Prediabetes by Both Criteria	Prediabetes OGTT only	Prediabetes HbA1c Only	*P*-Value
Number	100	150	146	50	
FPG (mg/dl)	5.2 ± 0.03	6.0 ± 0.06	5.9 ± 0.04	5.3 ± 0.04	< 0.0001
2-h PG (mg/dl)	5.7 ± 0.1	8.0 ± 0.2	7.2 ± 0.2	5.9 ± 0.1	< 0.0001
HbA1c (%)	6.00 ± 0.02	5.31 ± 0.03	5.35 ± 0.02	5.91 ± 0.03	< 0.0001
Triglycerides (mM)	1.41 ± 0.08	1.72 ± 0.07	1.68 ± 0.09	1.62 ± 0.10	< 0.05
HDL (mM)	1.21 ± 0.03	1.08 ± 0.02	1.09 ± 0.02	1.05 ± 0.03	NS
HOMA-IR	3.0 + 0.3	4.1 + 0.4	3.1 ± 0.3	3.6 ± 0.3	< 0.05
Beta Cell Function	55 ± 1	33 ± 1	34 ± 1	51 ± 2	< 0.0001
Stern Score	8.3 ± 0.6	22.5 ± 1.4	16.1 ± 11	9.8 ± 0.9	< 0.0001
% MS	20	58	62	20	< 0.0001

*FPG* fasting plasma glucose, *2-h PG* 2-h plasma glucose, *MS* metabolic syndrome

Lastly, the prevalence of metabolic syndrome was 3-fold greater in subjects with prediabetes than in NGT subjects according to the OGTT criteria, while it was similar in subjects with prediabetes and NGT according to HbA1c criteria.

## Discussion

The results of the present study demonstrate that the concordance between the two sets of criteria (OGTT and HbA1c) utilized to identify subjects at high future T2DM risk, i.e. prediabetes, is poor in Qatari subjects. Only 56% of subjects were diagnosed with NGT or prediabetes with both sets of criteria. The other 44% were diagnosed with prediabetes with one set of criteria and NGT with the other. The disagreement between OGTT and HbA1c was primarily due to lower sensitivity of HbA1c in identifying all subjects with IFG and/or IGT. These results are consistent with several previous studies in other ethnic groups [13–18] which also demonstrated low concordance between HbA1c and OGTT in diagnosing prediabetes, and the high risk subjects identified by each set of criteria are distinct. Further, subjects with prediabetes identified with the OGTT alone had more sever metabolic profile than subjects identified with HbA1c alone, and are more likely to progress to T2DM (Table 4). Suggesting that in this population, the OGTT is a better tool in identifying subjects at high risk of T2DM, i.e. prediabetes. The results of the present study also are consistent with previous study which demonstrated low sensitivity of HbA1c in identifying subjects with prediabetes diagnosed with OGTT in Arab Americans [24]. Accurate identification of subjects at increased future T2DM risk is important for diabetes prevention programs, reduces the number needed to treat and improves the cost effectiveness of the intervention program. The results of the present study demonstrate that, in the Qatari population, the OGTT identifies a larger number of subjects with prediabetes than the HbA1c, i.e. higher sensitivity. Stated otherwise, many IFG and/or IGT subjects who are at increased diabetes risk and who would have benefited from the intervention program have an HbAlc < 5.7% and, thus, would not have been identified using the HbA1c criteria. These findings are consistent with previous diabetes prevention studies [6–8] in which mean baseline HbA1c in subjects diagnosed as prediabetes with the OGTT was ~ 5.7–5.9%, indicating that many subjects with an HbA1c < 5.7% have prediabetes and would benefit from an intervention program.

We and others [9, 10, 23, 25] previously have shown that OGTT criteria have more sensitivity and specificity in identifying subjects at increased future T2DM compared to HbA1c criteria. Because there was large discordance between the prevalence of prediabetes diagnosed with HbA1c versus OGTT criteria in the present study, and longitudinal data is unavailable in this population, we utilized diabetes prediction models [21] to identify subjects at increased risk of diabetes and compare the future risk of diabetes in subjects diagnosed with each set of criteria. Further, many previous intervention studies have demonstrated that subjects at high risk of diabetes manifest impaired beta cell function long before T2DM is evident [10, 23], and the impairment in beta cell function was the strongest predictor of future T2DM risk. Because of lack of “gold standard” for identification of who is really at increased future T2DM risk, we utilized the risk score, and impairment in beta cell function as “surrogates” to compare the actual diabetes risk in subjects diagnosed with prediabetes according to OGTT and HbA1c criteria. The results of the present study have demonstrated that the OGTT is a better tool to identify subjects with higher score of Stern model (Table 3) and lower beta cell function (Table 4) than HbA1c. In other words, a subject with Hb1Ac < 5.7% who has IFG/IGT manifest greater chance of having high score in Stern model for future T2DM and impaired beta cell function than subject who has NGT (according to OGTT) and HbA1c = 6.0%.

Our results demonstrate that beta cell function in subjects diagnosed with prediabetes based upon the HbA1c (i.e. 5.7–.6.4%) but with NGT based on the OGTT is comparable to that in NGT subjects (Table 4), suggesting that relying on the HbA1c alone to identify high risk individuals will result in the inclusion of many subjects with normal beta cell function who actually are at low future T2DM risk (i.e., false positives). These results are consistent with our previous findings in Mexican Americans [24].

Third, many previous studies [25, 26] have demonstrated that subjects who fulfill the criteria of the metabolic syndrome manifest greater risk of cardiovascular disease (CVD) than subjects without the metabolic syndrome. The results of the present study demonstrate that prediabetes diagnosed with the OGTT is a stronger predictor of the metabolic syndrome and, therefore, subsequent CVD risk, than the HbA1c alone. Because the primary aim of diagnosing prediabetes is to identify the subgroup of subjects who are at increased future risk for T2DM and CVD, these findings indicate that, in Qatari subjects, the OGTT should be favored over the HbA1c for the identification of subjects with prediabetes.

We previously [27, 28] demonstrated that the 1-h plasma glucose concentration during the OGTT is the strongest predictor of future T2DM risk. Unfortunately, the 1-h plasma glucose concentration was not measured in the present study. The cross sectional nature of the study is another limitation. Future recall of the participants in the present cohort for a repeat OGTT will provide longitudinal information about the predictive power of the HbA1c and OGTT in Qatari individuals.

Other limitations of the study include: first, the study has cross sectional design without longitudinal follow-up. Second, only single OGTT was performed to diagnose glucose tolerance. Third, HOMA-B was utilized to measure beta cell function.

## Conclusion

In this high risk population for T2DM, the OGTT is more sensitive tool than HbA1c for the identification of subjects with prediabetes. The OGTT and HbA1c identify distinct populations as having high risk of diabetes. However, subjects identified as high risk of diabetes with the OGTT alone have more severe metabolic phenotype than those identified with HbA1c. Thus, OGTT should be favored over HbA1c in Qatar Diabetes Strategy to screen high risk individuals for intervention programs to reduce their future T2DM risk.

## Data Availability

The datasets used and/or analyzed during the current study are available from the corresponding author on reasonable request.

## Abbreviations

ADA: American Diabetes Association
CVD: Cardiovascular disease
FPG: Fasting plasma glucose
HbA1c: Glycated hemoglobin
HOMA: Homeostasis model assessment
IFG: Impaired fasting glucose
IGT: Impaired glucose tolerance
NGT: Normal glucose tolerance
T2DM: Type 2 diabetes mellitus
